# Comparative Analysis of Pediatric and Adult Mastocytosis: Clinical Presentation, Triggers, and Treatment Patterns from a Tertiary Care Registry

**DOI:** 10.3390/children13010141

**Published:** 2026-01-19

**Authors:** Sundus M. NoorSaeed, Roy Khalaf, Athari Alenezi, Eviatar Fields, Connor Prosty, Abdulaziz S. Alrafiaah, Barbara Miedzybrodzki, Elena Netchiporouk, John Sampalis, Michael Fein, Moshe Ben-Shoshan

**Affiliations:** 1 Department of Pediatrics, Faculty of Medicine, King Abdulaziz University, Jeddah 21589, Saudi Arabia; 2 Department of Pediatrics, Division of Allergy and Clinical Immunology and Dermatology, Montreal Children’s Hospital, Montreal, QC H3H 1P3, Canada; 3 Faculty of Medicine, McGill University, Montreal, QC H3A 2B4, Canada; 4 Division of Dermatology, McGill University Health Centre, Montreal, QC H4A 3J1, Canada; 5 Department of Pediatrics, College of Medicine, Majmaah University, Majmaah 11952, Saudi Arabia; 6 Division of Surgical Research, Surgical Epidemiology, McGill University, Montreal, QC H3A 2B4, Canada; 7 Division of Adult Allergy & Clinical Immunology, McGill University Health Centre, Montreal, QC H4A 3J1, Canada

**Keywords:** mastocytosis, cutaneous, systemic, tryptase, adults, pediatrics

## Abstract

**Background:** Mastocytosis is a rare hematologic disorder, classified into cutaneous mastocytosis (CM) and systemic mastocytosis (SM). Understanding age-related differences in presentation and management is essential for individualized care. **Methods:** Data from patients recruited from the Montreal Children’s and Montreal General Hospitals between 2015 and 2024 were analyzed. Descriptive statistics were employed to present patient demographics, clinical characteristics, and medication usage. Statistical analyses included Fisher’s exact test for categorical variables and *t*-tests or non-parametric equivalents for continuous variables. **Results:** A total of 63 patients were included, comprising 39 children and 24 adults. Children had a median age of 1.9 years, while adults had a median age of 49.3 years. CM was exclusively prevalent in children (100.0%), while SM was more common in adults (45.8%). Adults with SM had a significantly higher median age than CM (49.4 versus 44.7 years, respectively, *p* = 0.03). Epinephrine use was more frequent in adult SM patients (36.4% versus 0%, respectively, *p* = 0.03). No pediatric patients required epinephrine for symptom control. **Conclusions:** This study highlights important clinical differences between pediatric and adult mastocytosis. CM was more common in children while SM predominated in adults and was associated with greater flare severity and higher tryptase levels.

## 1. Introduction

Mastocytosis is a heterogenous group of disorders characterized by the abnormal accumulation of mast cells in various tissues [1]. Mast cells are immune cells involved in allergic response and tissue repair [2]; however, their dysregulated proliferation and activation in mastocytosis can result in a broad range of symptoms, from skin lesions to severe anaphylaxis [2]. Mastocytosis is classified primarily into cutaneous mastocytosis (CM), where mast cell proliferation is confined to the skin, and systemic mastocytosis (SM), in which mast cells infiltrate internal organs such as the bone marrow, liver, spleen, and gastrointestinal tract [3]. The prevalence of mastocytosis is estimated to be 10 per 100,000 adult population in the United States [4], although varying estimations have been reported in the literature [5,6]. Up to this point, prevalence estimates for pediatric mastocytosis have not been reported.

Diagnosis for CM is based mainly on clinical presentation according to criteria defined by the World Health Organization (WHO) [7] and is supported by histopathological examination of skin lesions [8]. In suspected SM, additional confirmatory tests are required, including bone marrow aspiration, serum tryptase levels, and analysis for C-KIT mutations [9]. Understanding how CM and SM presentations differ between pediatric and adult populations is crucial for appropriate risk stratification, therapeutic decision-making, and patient education. The objective of this study is to evaluate and compare clinical presentation, flare-up triggers, and treatment strategies in CM and SM in children versus adults.

## 2. Methods

Data for this study were collected through the Mastocytosis Registry, a comprehensive database encompassing both pediatric and adult patients who visited the dermatology and allergy clinics at the Montreal Children’s Hospital (MCH) and Montreal General Hospital (MGH). We collected data prospectively and retrospectively using a standardized data entry form. Informed written consent was obtained from all participating patients.

Data were collected on age, sex, clinical presentation, history of co-morbidities, including allergic diseases and autoimmune diseases, management of mastocytosis (including antihistamines, steroids, and other medications), triggers of flares, and family history of co-morbidities. Diagnoses of mastocytosis types, including CM and SM, were specified based on the diagnosis of the treating allergist/dermatologist. Blood tests drawn included complete blood count (CBC), baseline tryptase, and liver function tests (LFTs). SM diagnoses in this registry reflect the treating allergist’s or dermatologist’s diagnosis at the time of registry entry, based on supportive laboratory or histopathologic findings when required, based on the World Health Organization (WHO) criteria for different forms of mastocytosis [10]. These diagnoses were informed by available clinical features, laboratory data, and histopathologic findings. Additional staging investigations for suspected SM, such as bone marrow biopsy and peripheral blood or tissue testing for the KIT D816V mutation, were performed at the discretion of the treating specialist and were not uniformly required for registry inclusion. Consequently, comprehensive staging data were not available for all patients, particularly those recruited through the national patient association and followed outside of our institutions. The presence of IgE-mediated allergic disease alone did not determine mastocytosis classification and was recorded separately as a comorbidity.

### Statistical Analysis

Descriptive statistics were employed to present patient demographics, clinical characteristics, and medication usage. Statistical analyses were performed using R version 4.2.3 for macOS 10.3 (R Foundation for Statistical Computing, Vienna, Austria). Categorical data were expressed as percentages, while continuous data were reported as medians with interquartile ranges (IQR). Given the small sample size and heterogeneity in age, distributions of continuous variables were assessed for normality and were found to be non-normal. Accordingly, non-parametric statistical tests were used for the analysis of quantitative data. Fisher’s exact test was used to compare categorical variables, particularly in the context of small sample sizes.

## 3. Results

From 2015 to 2024, 63 participants with mastocytosis presenting to the MCH and MGH were recruited in our registry, including 39 children and 24 adults. Among children, the median age was 1.9 years old (interquartile range (IQR), 1.2–3.8), and 66.6% were males. Among adults, the median age was 49.3 years (IQR, 38.3–56.8), and 33.3% were males.

### 3.1. Pediatric Mastocytosis

Among 39 children presenting with mastocytosis, all patients presented with CM. Within that cohort, three patients (7.7%) were asthmatic, and two (5.1%) had atopic dermatitis. (Table 1). Ten patients with CM presented with urticaria pigmentosa, one with bullous mastocytosis, four with mastocytoma, one with polymorphic cutaneous mastocytosis, and the remaining were unknown.

Among the 39 children, 15 (38.5%) received antihistamine treatment, while eight (20.5%) were treated with steroids. Nearly half (46.2%) did not receive any treatment. The main physical triggers associated with flares included sun exposure (33.3%), exercise (23.1%), and water (23.1%) (Table 1). Food accounted for 15.4% of flares, mainly strawberries (10.3%), with unknown triggers accounting for 64.1% of flare-ups.

The median number of flare-ups per year was 2.0 (IQR 0.0–4.0) across the cohort. For flare-up management, 14 patients (35.9%) were treated with antihistamines, with eight (20.5%) being first-generation and one (2.6%) second-generation. Within our cohort, six (15.4%) received topical steroids. No patients required epinephrine for flare-ups.

### 3.2. Adult Mastocytosis

Among the adult patients, 13 (54.2%) were diagnosed with CM, while 11 (45.8%) had SM. The median age was higher in SM compared to CM (49.4 versus 44.7, respectively, *p* = 0.03). No significant differences were noted between groups in terms of sex distribution, asthma prevalence, allergic rhinitis, or atopic dermatitis (Table 2). Among the adults with CM, seven presented with urticaria pigmentosa, four with telangectasia macularis eruptiva perstans, one with mastocytoma, one with diffuse CM, and the remaining were unknown. Follow-up information was available for 5 of the 11 adult patients with SM; all had undergone bone marrow biopsy and were positive for the D816V KIT variant. Two of these patients reported an initial diagnosis of cutaneous mastocytosis prior to progression to systemic disease.

Antihistamines were the most used therapy (79.2%) with a significantly higher prevalence in SM compared to CM (100.0% versus 61.5%), respectively, *p* = 0.02. First-generation antihistamines were used in six patients (25.0%), and second-generation antihistamines in five (20.8%). Steroids were used by 23.1% of CM patients and 27.3% of SM patients.

Sun exposure was reported as a flare-up trigger in 33.3% of patients and was significantly more frequent in SM compared to CM (63.6% versus 7.7%, respectively, *p* < 0.01). Exposure to exercise (50.0%), pressure (16.7%), and cold (33.3%) was also noted, though no significant differences were found between CM and SM.

Food-related flare-ups were reported in more than a third of cases (37.5%), particularly eggplant (12.5%). Alcohol was reported to trigger flares in almost half of the cases, antibiotics in 17%, and NSAIDs in less than 10% of cases. There were no significant differences regarding triggers between SM and CM. Patients reported that antihistamines were the main treatment used to control flare-ups (45.8%), followed by prednisone (20.8%) and epinephrine (16.7%). Epinephrine was significantly more used in SM compared to CM (36.4% versus 0%, *p* = 0.03). The median number of annual flare-ups was 4.0 (IQR 0.0–4.0) across both groups, with no difference between SM and CM. Baseline tryptase level was higher in SM compared to CM (26.0 versus 10.4, respectively, *p* = 0.02). No significant differences in AST, ALT, absolute eosinophils, and neutrophils were noted (Table 2).

Among the CM cohort, 13 adults (25.0%) and 39 pediatric patients (75.0%) were included. All adults with cutaneous presentation reported presentation consistent with monomorphic maculopapular cutaneous mastocytosis (MPCM). No significant differences were noted between groups in terms of sex distribution, asthma prevalence, or atopic dermatitis (Table 3). Regarding daily treatment, antihistamines were the most used therapy (61.5%). Steroids were used by 23.1% of adults and 20.5% of children. Absence of treatment was more common in children compared to adults (46.2% versus 15.4%, respectively, *p* < 0.01).

Vibration exposure was reported exclusively in adults (15.4% versus 0.0%, *p* = 0.01). There were no differences between the two groups regarding exposure to pressure, cold, or water. Regarding flare-up triggers, food-related reactions were the most commonly reported in adults (37.5%), mainly with eggplant in adults versus strawberry in children. Alcohol was reported as a trigger only in adults (38.5% versus 0.0%, *p* < 0.01).

For flare-up treatment, antihistamines were most frequently administered (34.6%), followed by steroids (13.5%). Second-generation antihistamines were significantly more frequently used in adults (23.1% versus 2.6%, respectively, *p* = 0.04). The median number of annual flare-ups was 4.0 (IQR 0.0–4.0) in adults and 2.0 (IQR 0.0–4.0) in pediatric patients (*p* = 0.26).

## 4. Discussion

This study is the largest Canadian study to compare children and adults with mastocytosis, their clinical presentations, symptomatology, and management.

In the pediatric population, CM was found to be the most prevalent subtype of mastocytosis, affecting 100.0% of our pediatric cohort, consistent with existing literature [11]. In contrast, SM was more frequently diagnosed in adults, with a significantly higher median age in our cohort of adults with SM compared to those with CM (49.4 versus 44.7 years, respectively, *p* = 0.03). This age-related difference in subtype distribution may reflect distinct underlying disease mechanisms. SM is commonly associated with activating mutations in the KIT gene, leading to abnormal mast cell proliferation [12]. This c-KIT mutation gene was found in 80% of adult mastocytosis patients [13] but has been reported in varying prevalences in children, going from 0% to 83% [14]. This variability suggests that pediatric CM may not always be driven by KIT mutations. Differences in KIT expression and the local mast cell environment may contribute to diverse disease trajectories, with pediatric CM more likely to regress and adult forms tending to persist [15].

SM was associated with more severe clinical features in adults. Indeed, in our cohort, epinephrine was more frequently administered in adult SM patients compared to adults with CM (36.4% versus 0%, respectively). This suggests that SM patients had a higher risk of systemic and potentially life-threatening reactions. Additionally, baseline tryptase levels were significantly elevated in adult SM patients compared to those with CM, reinforcing its established role. Persistently elevated serum tryptase levels (>20 ng/mL) are a minor diagnostic criterion of SM defined by the World Health Organization (WHO) criteria [7]. This elevated tryptase not only supports diagnosis but also serves as a marker for mast cell burden, correlating with increased risk of anaphylaxis in patients with SM [16].

In our cohort, antihistamines were the most common treatment, followed by steroids, with consistent management between adults and children. First-generation antihistamines were used more frequently than second-generation options, aligning with recommendations from Cardet et al. [17] suggesting SM patients be treated with daily first-generation antihistamines. However, first-generation agents are associated with more side effects such as sedation [18], and recent studies suggest that the two groups have similar efficacy, although the safety profile is better for second-generation antihistamines [19]. Hence, we suggest reassessment of recommendations to advise on preferable use of second-generation antihistamines.

This study had some limitations. Firstly, the findings from this study come from a single center, limiting the generalizability of the findings to broader populations or different healthcare settings. Secondly, the relatively small sample size, particularly in the SM pediatric subgroup, precludes definitive conclusions. Our SM classifications represent a snapshot at the time of registry entry based on the treating specialist’s assessment, and comprehensive staging data were not available for all patients. This is particularly relevant for patients recruited from outside Quebec through the national patient association, as we did not have access to their complete hematology records. As such, some SM cases may have been underreported or incompletely characterized, and our prevalence estimates should be interpreted with this limitation in mind. Although we were not able to access medical records for all patients, discussions with patients reveal symptoms and presentations consistent with indolent macrocytosis (ISM) rather than more aggressive forms. In our cohort, baseline tryptase values were available for all adult patients; however, staging investigations were not uniformly performed. Further studies with sample size and long-term follow-up are needed to better understand the history, optimal management, and progression of mastocytosis across different age groups.

## 5. Conclusions

This study highlights important clinical differences between pediatric and adult mastocytosis. CM was more common in children, while SM predominated in adults and was associated with greater flare severity and higher tryptase levels. Understanding these differences is crucial for age-specific diagnosis, management, and follow-up. Further multicenter studies are warranted to refine care strategies.

## Figures and Tables

**Table 1:** General characteristics of pediatric cutaneous mastocytosis.

	Total Pediatric Mastocytosis N (%) N = 39
Median age at diagnosis (IQR)	1.9 (1.2–3.8)
Male sex	26 (66.7)
Asthma	3 (7.7)
Allergic rhinitis	0
Atopic dermatitis	2 (5.1)
**Regular treatment**	
Antihistamines	15 (38.5)
Steroids	8 (20.5)
Other	5 (12.8)
No treatment given	18 (46.2)
**History of exposure**	
Exposure to sun	13 (33.3)
Exposure to vibration	0
Exposure to exercise	9 (23.1)
Exposure to water	9 (23.1)
Exposure to pressure	6 (15.4)
Exposure to cold	5 (12.8)
Unknown	25 (64.1)
**Flare-up trigger**	
Antibiotics	1 (2.6)
Amoxicillin	1 (2.6)
**NSAIDs**	0
Other (Singulair, Paracetamol, Morphine)	2 (5.1)
Do not know	14 (35.9)
Alcohol YES	0
Alcohol NO	8 (20.5)
Alcohol—do not know	13 (33.3)
Peanut	0
Tree nut	0
Strawberry	4 (10.3)
Eggplant	1 (2.6)
Other food	6 (15.4)
Do not know	9 (23.1)
Minor trauma	8 (20.5)
Major trauma	0
Other trigger	8 (20.5)
No clear trigger	18 (46.2)
Number of flare-ups in a year	2.0 (0.0–4.0)
**Treatment of flare-ups**	
Antihistamines for flare-ups	14 (35.9)
First-generation	8 (20.5)
Second-generation	1 (2.6)
Steroids for flare-ups	6 (15.4)
Mometasone furoate	3 (7.7)
Desonide 0.05%	1 (2.6)
Emollient creams	1 (2.6)
Cromolyn	2 (5.1)
Omalizumab	2 (5.1)
Epinephrines	0
Other	4 (10.3)
No treatment	18 (46.2)

**Table 2:** Comparison of general characteristics between adult cutaneous and systemic mastocytosis.

	Adult Population Mastocytosis N (%) N = 24	Adult Cutaneous Mastocytosis N (%) N = 13	Total Adult Systemic Mastocytosis N (%) N = 11	*p* Values
Median age at diagnosis (IQR)	49.3 (38.3–56.8)	44.7 (37.0–52.8)	49.4 (45.7–58.5)	**0.03**
Male sex	8 (33.3)	5 (38.5)	3 (27.3)	0.56
Asthma	3 (12.5)	3 (23.1)	0	0.09
Allergic rhinitis	5 (20.8)	3 (23.1)	2 (18.2)	0.77
Atopic dermatitis	2 (8.3)	1 (7.7)	1 (9.1)	0.90
**Regular treatment**				
Antihistamines	19 (79.2)	8 (61.5)	11 (100.0)	**0.02**
Steroids	6 (25.0)	3 (23.1)	3 (27.3)	0.81
Other	11 (45.8)	6 (46.2)	5 (45.5)	0.97
No treatment given	2 (8.3)	2 (15.4)	0	0.17
**History of exposure**				
Exposure to sun	8 (33.3)	1 (7.7)	7 (63.6)	**<0.01**
Exposure to vibration	4 (16.7)	2 (15.4)	2 (18.2)	0.85
Exposure to exercise	12 (50.0)	5 (38.5)	7 (63.6)	0.22
Exposure to water	3 (12.5)	1 (7.7)	2 (18.2)	0.44
Exposure to pressure	7 (29.2)	3 (23.1)	4 (36.4)	0.48
Exposure to cold	8 (33.3)	3 (23.1)	5 (45.5)	0.25
No	4 (16.7)	1 (7.7)	3 (27.3)	0.23
Other	12 (50.0)	7 (53.9)	5 (45.5)	0.68
**Flare-up trigger**				
**Antibiotics**	4 (16.7)	2 (15.4)	2 (18.2)	0.86
Amoxicillin	1 (4.2)	0	1 (9.1)	0.27
Azithromycin	1 (4.2)	0	1 (9.1)	0.27
Ciprofloxacin	1 (4.2)	1 (7.7)	0	0.35
Penicillin	1 (4.2)	1 (7.7)	0	0.35
**NSAIDs**	2 (8.3)	1 (7.7)	1 (9.1)	0.90
Ibuprofen	1 (4.2)	0	1 (9.1)	0.27
Naproxen	1 (4.2)	1 (7.7)	0	0.35
Other (Cold and flu, ondansetron, cetirizine, codeine)	7 (29.2)	4 (30.8)	3 (27.3)	0.85
Alcohol YES	12 (50.0)	5 (38.5)	7 (63.6)	0.22
Alcohol NO	5 (20.8)	4 (30.8)	1 (9.1)	0.19
Peanut	0	0	0	N/A
Tree nut	0	0	0	N/A
Strawberry	1 (4.2)	1 (7.7)	0	0.35
Eggplant	3 (12.5)	2 (15.4)	1 (9.1)	0.64
Other food	9 (37.5)	3 (23.1)	6 (54.6)	0.11
Minor trauma	7 (29.2)	4 (30.8)	3 (27.3)	0.85
Major trauma	1 (4.2)	1 (7.7)	0	0.35
Other trigger	8 (33.3)	4 (30.8)	4 (36.4)	0.77
No clear trigger	7 (29.2)	5 (38.5)	2 (18.2)	0.28
Median number of flare-ups (IQR)	4.0 (4.0–4.0)	4.0 (0.0–4.0)	4.0 (0.0–4.0)	0.14
**Treatment of flare-ups**				
**Antihistamines for flare-ups**	11 (45.8)	4 (30.8)	7 (63.6)	0.11
First-generation antihistamines	6 (25.0)	1 (7.7)	5 (45.5)	0.06
Second-generation antihistamines	5 (20.8)	3 (23.1)	2 (28.6)	0.99
Steroids for flare-ups	5 (20.8)	1 (7.7)	4 (36.4)	0.08
Prednisone	5 (20.8)	1 (7.7)	4 (36.4)	0.08
Emollient creams	1 (4.2)	1 (7.7)	0	0.35
Epinephrines	4 (16.7)	0	4 (36.4)	0.08
Other	8 (33.3)	4 (30.8)	4 (36.4)	0.77
No treatment	5 (20.8)	4 (30.8)	1 (9.1)	0.19
**Laboratory values**				
Baseline tryptase, median (IQR)	11.1 (6.0–15.5)	10.4 (7.9–17.3)	26.0 (21.1–94.7)	**0.02**
AST	18.0 (16.0–21.0)	17.0 (16.0–20.0)	24.5 (21.9–27.6)	0.12
ALT	17.0 (14.0–20.8)	17.0 (14.0–20.0)	24.0 (22.0–30.5)	0.06
Absolute eosinophils	0.15 (0.10–0.19)	0.14 (0.11–0.17)	0.15 (0.075–0.35)	0.93
Absolute neutrophils	3.4 (2.2–4.4)	3.6 (2.2–4.4)	3.2 (2.0–4.4)	0.77

Reference ranges: Serum tryptase: <11.4 ng/mL; absolute eosinophil count: 0.0–0.5 × 10^9^/L; absolute neutrophil count: 1.5–7.5 × 10^9^/L. CM in adults vs. children.

**Table 3:** Comparison of general characteristics between adult cutaneous and pediatric cutaneous mastocytosis.

	Adult Cutaneous Mastocytosis N (%) N = 13	Pediatric Cutaneous Mastocytosis N (%) N = 39	*p* Values
Median age at diagnosis (IQR)	44.7 (37.0–52.8)	1.9 (1.2–3.8)	**<0.01**
Male sex	5 (38.5)	26 (66.7)	0.10
Asthma	3 (23.1)	3 (7.7)	0.16
Allergic rhinitis	3 (23.1)	0	**<0.01**
Atopic dermatitis	1 (7.7)	2 (5.1)	0.99
**Regular treatments**			
Antihistamines	8 (61.5)	15 (38.5)	0.20
Steroids	3 (23.1)	8 (20.5)	0.99
Other	6 (46.2)	5 (12.8)	**0.02**
No treatment given	2 (15.4)	18 (46.2)	**0.04**
**History of exposure**			
Exposure to sun	1 (7.7)	13 (33.3)	0.15
Exposure to vibration	2 (15.4)	0	**0.01**
Exposure to exercise	5 (38.5)	9 (23.1)	0.30
Exposure to water	1 (7.7)	9 (23.1)	0.42
Exposure to pressure	3 (23.1)	6 (15.4)	0.67
Exposure to cold	3 (23.1)	5 (12.8)	0.39
No	1 (7.7)	9 (23.1)	0.42
Other	7 (53.9)	14 (35.9)	0.33
**Flare-up triggers**			
**Antibiotics**	2 (15.4)	1 (2.6)	0.15
Amoxicillin	0	1 (2.6)	0.99
Ciprofloxacin	1 (7.7)	0	0.25
Penicillin	1 (7.7)	0	0.25
**NSAIDs**	1 (7.7)	0	0.25
Advil	0	0	N/A
Naproxen	0	0	N/A
Other (Cold and flu, ondansetron, Cetirizine, codeine)	4 (30.8)	2 (5.1)	**0.02**
Alcohol YES	5 (38.5)	0	**<0.01**
Alcohol NO	4 (30.8)	8 (20.5)	0.61
Peanut	0	0	N/A
Tree nut	0	0	N/A
Strawberry	1 (7.7)	4 (10.3)	0.47
Eggplant	2 (15.4)	1 (2.6)	0.15
Other food	3 (23.1)	6 (15.4)	0.67
Minor trauma	4 (30.8)	8 (20.5)	0.47
Major trauma	1 (7.7)	0	0.25
Other trigger	4 (30.8)	8 (20.5)	0.47
No clear trigger	5 (38.5)	18 (46.2)	0.75
Median number of flare-ups (IQR)	4.0 (0.0–4.0)	2.0 (0.0–4.0)	0.26
**Treatment of flare-ups**			
Antihistamines for flare-ups	4 (30.8)	14 (35.9)	0.68
First-generation antihistamines	1 (7.7)	8 (20.5)	0.42
Second-generation antihistamines	3 (23.1)	1 (2.6)	**0.04**
Steroids for flare-ups	1 (7.7)	6 (15.4)	0.66
Mometasone furoate	1 (7.7)	1 (2.6)	0.47
Cromolyn creams	0	1 (2.6)	N/A
Omalizumab	0	1 (2.6)	N/A
Epinephrines	0	1 (2.6)	N/A
Other	4 (30.8)	4 (10.3)	0.09
No treatment	4 (30.8)	18 (46.2)	0.52
**Laboratory values**			
Baseline tryptase	10.4 (7.9–17.3)	5.5 (4.5–10.1)	0.46

Reference ranges: Serum tryptase: <11.4 ng/mL.

## Data Availability

The data that support the findings of this study are not publicly available due to privacy reasons but are available from Roy Khalaf upon request. Participants did not provide consent for their data to be shared publicly at the time of collection. As such, the dataset cannot be made publicly available. Researchers may contact the corresponding author for limited access under specific conditions.

## Abbreviations

CM (Cutaneous Mastocytosis), SM (Systemic Mastocytosis), IQR (Interquartile range), CI (Confidence interval), OR (Odds ratio).
